# Medical Hydrogeology of Asian Deltas: Status of Groundwater Toxicants and Nutrients, and Implications for Human Health

**DOI:** 10.3390/ijerph13010081

**Published:** 2015-12-26

**Authors:** Mohammad A. Hoque, Adrian P. Butler

**Affiliations:** Department of Civil and Environmental Engineering, Imperial College London, South Kensington, London SW7 2AZ, UK; a.butler@imperial.ac.uk

**Keywords:** groundwater, nutrients, Asia, delta, medical hydrogeology, arsenic, minerals

## Abstract

Drinking water, a fluid primarily for human hydration, is also a source of mineral nutrients. Groundwater, a drinking water source for more than 70% of inhabitants living in Asian deltas, has received much attention because of its naturally occurring arsenic, but the linkage of arsenic toxicity with other water constituents has not been studied. In addition, although nutrients are generally provided by food, in under developed rural settings, where people subsist on low nutrient diets, drinking-water-nutrients may supply quantities vital to human health thereby preventing diseases. Here, we show, using augmented datasets from three Asian deltas (Bengal, Mekong, and Red River), that the chemical content of groundwater is such that in some areas individuals obtain up to 50% or more of the recommended daily intake (RDI) of some nutrients (e.g., calcium, magnesium, iron) from just two litres of drinking water. We also show some indications of a spatial association of groundwater nutrients and health outcome using demographic health data from Bangladesh. We therefore suggest that an understanding of the association of non-communicable disease and poor nutrition cannot be developed, particularly in areas with high levels of dissolved solids in water sources, without considering the contribution of drinking water to nutrient and mineral supply.

## 1. Introduction

Water is essential for all forms of life and plays an essential role in maintaining fluid and electrolyte homeostasis in humans. Sustaining hydration is necessary for thermoregulation and normal bodily function. Drinking water, although often rich in minerals, has rarely been considered in terms of its ability to supply essential mineral nutrients e.g., [1]. It can be hypothesised that groundwater, whose nutrient composition is controlled by geology and geochemistry, could confer some health benefits and might mitigate some diseases. Populations living in resource-poor settings, who subsist on low nutrient diets, may suffer from nutrient deficiency and the associated health consequences. In such setting drinking water rich in mineral content can provide a significant amount of the recommended daily nutrient requirement. However, this contribution has received scant attention. Groundwater chemistry, and hence mineral content, varies spatially, on a range of scales from local to global. We postulate that this varied, and often unrecognised, mineral content could help some (where intake via food is insufficient) to reach the recommended daily level of nutrients, and hence avoid nutrient deficiency related consequences [2]. Exploring this contribution is also important, as it could result in adverse health consequences when nutrient rich groundwater is exchanged for alternative sources (e.g., harvested rainwater or reverse osmosis water) with lower nutrient levels.

Medical geology is a branch of geology that studies the relationship between health and geological conditions e.g., [3,4,5]. This relationship can be both positive and negative. In a similar manner “medical hydrogeology”, investigates the relationship between groundwater and health. Historically the focus has been on the diseases associated with certain elements (e.g., arsenic, fluoride, manganese *etc.*). Rarely has there been consideration of the positive health effects of mineral-nutrients (e.g., calcium, magnesium, iron *etc.*). Medical hydrogeology, however, should not only consider the harmful elements but also those that influence the toxicity of harmful elements and those (e.g., bicarbonate, pH, iron *etc*.) that alter the groundwater characteristics during storage and treatment. For example, fluoride is harmful in drinking water if it is at a concentration above 1.5 mg/L. However, toxicity of fluoride can be reduced if the the groundwater and/or diet contain high levels of calcium e.g., [6,7].

**Figure 1:**
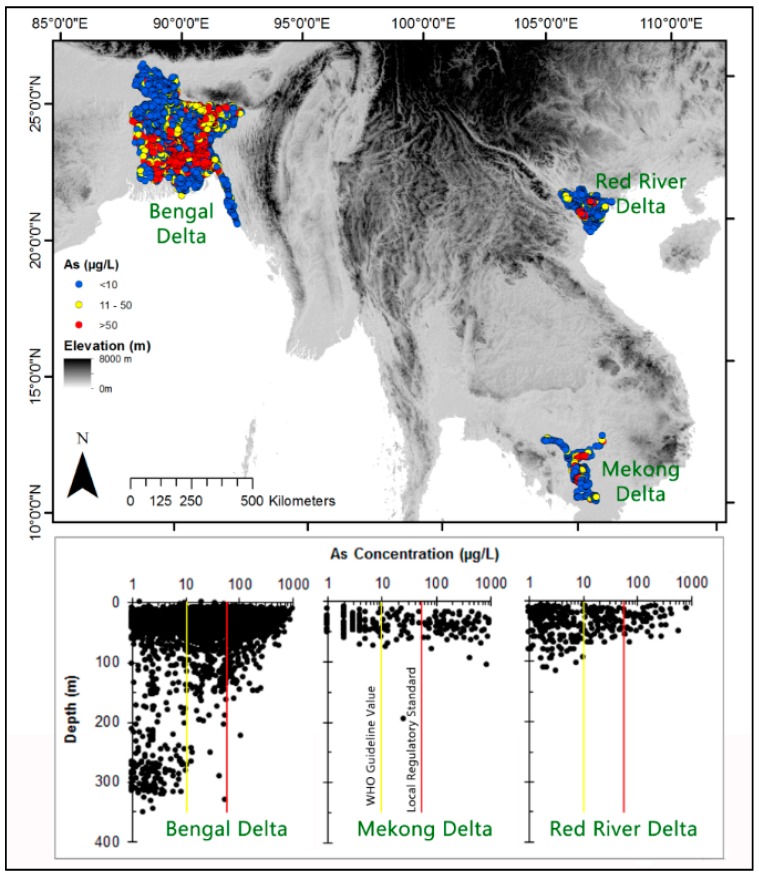
Location of the East and Southeast Asian deltas and coverage of data [19,22,35,36,37] used in this study. Elevation (based on [38]) variation across the region indicate the topography and landscape. Depths covered by the dataset for a whole range of ions are illustrated by As.

Around 300 million people living in Asian deltas rely on groundwater for their water supply. In these areas a nutrient rich diet is not widely affordable and many live on limited cereal based diets, which can lead to micronutrient malnutrition. At present >2 billion of people on the globe suffer from this, and many of them live in these delta areas [8]. We have compiled a comprehensive data set (Figure 1) of groundwater chemistry from three Asian deltas (Bengal, Mekong and Red River), and analyse the data to demonstrate the “unrecognised” nutrient status of groundwater, with particular reference to their fraction of total dietary RDI.

We also carried out a preliminary exploratory study in Bangladesh to assess the possible health benefits of these groundwater nutrients at a national scale. We would like to relate drinking water and health within a framework of “medical hydrogeology”, which links the hydrogeology of an area with certain diseases and health benefits due to presence, or shortage of certain nutrients in groundwater.

## 2. Hydrogeology and Health

### 2.1. Hydrogeological Settings

Geologically, Asia is very diverse, characterised by highlands and mountainous terrains of igneous and metamorphic rocks and thick piles of unconsolidated to semi-consolidated sediments in mega-deltas formed along the coasts by the rivers draining the highlands and mountains, e.g., [9]. Asian rivers carry billions of tonnes of suspended loads, and a significant fraction is deposited to build these deltas [10]. These sediments are physically, but not generally chemically, weathered. This makes them susceptible to rock-water interactions—a process that can lead to a high mineral content in the groundwater in these areas.

The current landforms in these deltas have been determined, during Late Quaternary time, by geological modifications [10,11,12,13,14]. All these deltas are characterised by Holocene sediments of fluvio-deltaic origin in the near-surface to a depth up to *ca.* 50 m. The sediments are sand, silt and clay with occasional gravel, and overlay older sediments of a similar origin. All these deltas experienced periodic eustatic changes leading to episodes of (*ca.* 100 ka) sub-aerial weathering and (*ca.* 10–20 ka) deposition [15]. The sub-aerial weathering was coupled with a lower (paleo) water table and a *ca.* 120 m drop in global sea level leading to groundwater flushing during the low stands. By contrast, at high stands increased sedimentation leads to the formation of new aquifers on top of the older well flushed, and often sub-aerially weathered, desposits [16,17]. The dissolved ions of the well flushed aquifers are significantly lower than those of the recent aquifers.

In recent decades the discovery of, mostly naturally occurring, arsenic in shallow groundwater has led to a number of hydrogeological studies of these aquifers e.g., [15,18,19,20,21,22]. These have shown that inland groundwater at shallow (*ca.* 150 mbgl) depths is polluted by naturally occurring arsenic e.g., [23], whereas, in coastal areas they are pervasively impacted by natural salinity [24]. In contrast, deep groundwater, both inland and coastal, is generally fresh and arsenic “safe” [25,26].

### 2.2. Groundwater and Health Impact

Although the use of groundwater as a source of drinking water in Asia has a long history, it was only during the period between 1970 and 2000 that it markedly expanded to its current extent [27,28]. The reason for this increased usage was to avoid vector borne cholera and diarrheal diseases (one of the top causes of morbidity and mortality in the region and particularly associated with surface water sources) [28,29]. The move significantly reduced vector borne diseases in the region, but inadvertently introduced naturally occurring arsenic, which is particularly extensive in Asian deltas, the impact of which was discovered during the 1990s. This has been termed the “largest mass poisoning of a population in history” [30]. A study in 2010 estimated that around 20% of all the deaths in Bangladesh are linked to the arsenic poisoning [31].

In addition to the widespread effects of high arsenic concentrations in these deltaic groundwaters, there are other potential health impacts from high levels of dissolved ions present in these waters. In particular, manganese, which, although an essential micro-nutrient, at high concentrations can hinder mental development in children [32]. Also, excessive sodium concentrations, such as occur in the coastal groundwaters in these deltaic areas, have been linked with hypertension and preeclampsia in pregnant women [33,34].

## 3. Methods and Materials

### 3.1. Data

A comprehensive water quality dataset has been compiled for 5256 tube-wells using published literature [19,22,35,36,37] for three Asian deltas, Bengal, Mekong and Red River (Figure 1). Most of the wells have a full range of chemical composition, including trace elements. However, in view of the focus on health impacts, the key determinands considered are: arsenic (As), bicarbonate (HCO_3_), calcium (Ca), fluoride (F), iodine (I), iron (Fe), magnesium (Mg), manganese (Mn), nitrate (NO_3_), potassium (K), selenium (Se), sodium (Na), uranium (U), vanadium (V), and zinc (Zn). Analytical procedures and the actual data can be found in the cited literature sources.

### 3.2. Data Treatment and Assumptions

The RDI for different nutrients vary according to age group, as well as for special groups (e.g., pregnant women), however, for simplicity a fixed conservative value e.g., [39] is considered here for each mineral nutrient: Ca (800 mg/d), F (4 mg/d), Fe (14 mg/d), I (150 µg/d), K (3000 mg/d), Mg (300 mg/d), Mn (2 mg/d), Na (2000 mg/d), Se (55 µg/d) and Zn (10 mg/d).

For proper bodily function and hydration a person needs around 3.5 litres of fluid. Some of this comes from food, but a significant part of comes from drinking water and other drinks. A conservative assumption is made that a person living in an Asian delta drinks two litres of water from the same well, though some studies, e.g., [40,41], have shown it can be up to 3.5 litres. Bioavailability of nutrients varies, and depends on a number of factors (e.g., chemical form, food matrix, age, gender, nutrient status *etc.*) [42]. However, again for simplicity, it is assumed that all the elements present in drinking water are readily bioavailable.

### 3.3. Groundwater Nutrients and Health—Bangladesh Case Study

Drinking water in Bangladesh, which occupies most of the Bengal delta, is almost entirely (*ca.* 95%) sourced from groundwater [43]. We have, therefore, used Bangladesh as an exemplar to explore the relationship between health effects and the presence of mineral nutrients in groundwater. The British Geological Survey (BGS) in collaboration with the Department of Public Health and Engineering (DPHE) have made available a database of chemical composition of 3805 tubewells from across Bangladesh [22], which was used for comparison with health (meta) data. The demographic health survey data on the probability of stunting in children under the age of five years, the prevalence of rickets and other leg deformities, anaemia patients, and prevalence of arsenicosis patients from various sources were collected as metadata or as images (data sources are given as they appear in the text). The associations between the spatial prevalence of different health conditions are compared with the respective spatial patterns in the water quality data.

## 4. Results

### 4.1. Status of Macro-Minerals

**Figure 2:**
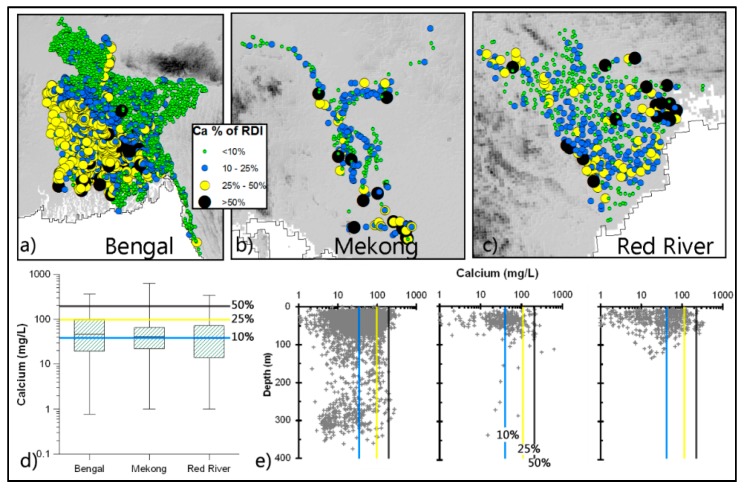
Concentration of Ca in groundwater in relation to RDI. Spatial (**a**–**c**) and vertical (**e**) variations of well-water Ca, and box plots (**d**) showing basic statistical (minimum, maximum, median, lower quartile and upper quartile) properties of well-water Ca concentration.. Boxes represent IQR (25th and 75th percentile) with the line at the median.

**Figure 3:**
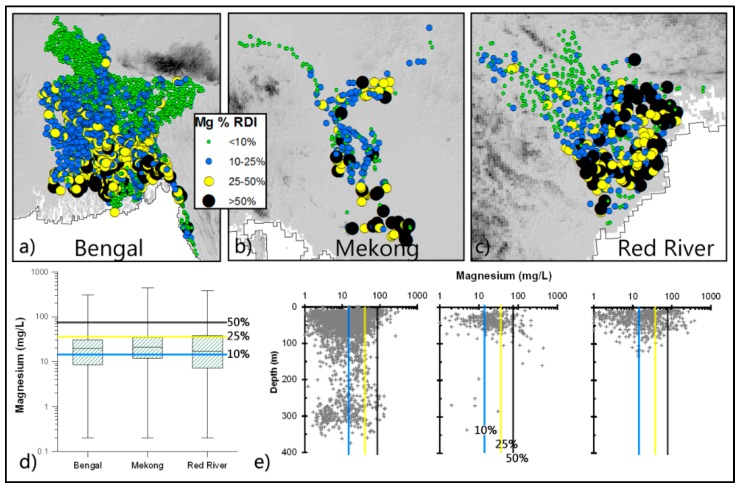
Concentration of Mg in groundwater in relation to RDI. Spatial (**a**–**c**) and vertical (**e**) variations of well-water Mg, and basic statistical properties (as Figure 2) of well-water Mg are shown in the box plots (**d**).

The human body needs relatively large amounts of macro-minerals (Ca, Mg, Na, K, P, Cl and S), some of which can be found in groundwater, as outlined below:

*Calcium (Ca) and Magnesium (Mg):* Significant amounts, up to 50% RDI of Ca and Mg, can be derived from groundwater in some areas (Figure 2 and Figure 3). Around 23% of wells provide 25% or more of the RDI of Ca, and 15% for Mg. Most of these high Ca and Mg wells are located in the south-west and southern parts of the deltas. There is no clear depth-wise trend for these concentrations.

**Figure 4:**
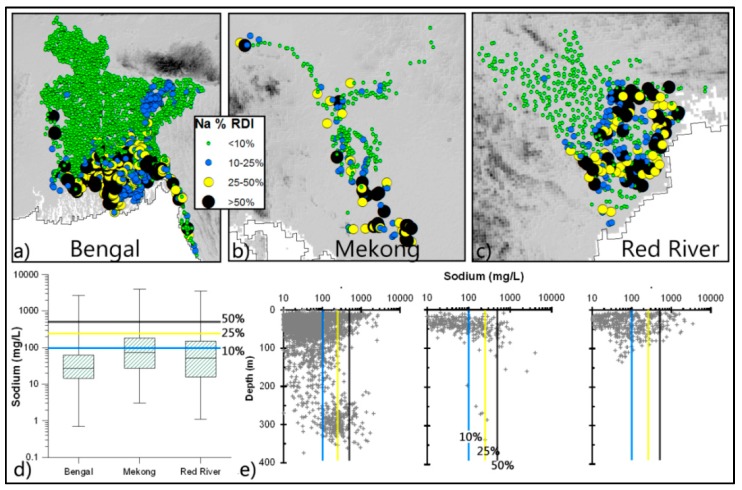
Concentration of Na in groundwater in relation to RDI. Spatial (**a**–**c**) and vertical (**e**) variations of well-water Na; and basic statistical properties (as Figure 2) of well-water Na are shown in the box plots (**d**).

*Sodium (Na):* Although the human body needs Na, it is generally assumed that this is met from food and added salt. However, in coastal deltas a significant amount of the daily Na requirement (as well as chloride—though not considered here) can be obtained from drinking water (Figure 4). Most wells analysed (*ca.* 80%) provide less than 10% of RDI, however around 4% wells, particularly in coastal areas, contain more than 500 mg/L of Na, which is more than 50% of RDI. The EPA guidance level for Na in drinking water is 20 mg/L and 65% of the wells exceed this concentration. In contrast, there is no health-based guideline provided by the WHO, however, it does, on the grounds of taste, recommend a limit of 200 mg/L. Around 12% of the wells have concentrations above this value.

**Figure 5:**
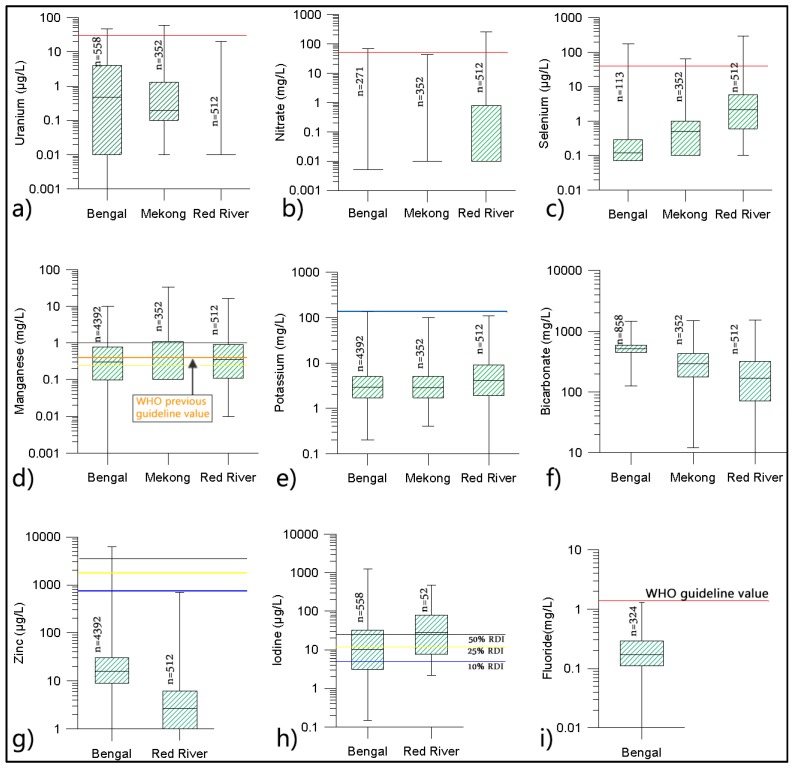
Basic statistical properties (minimum, maximum, median, lower and upper quartile) for various groundwater ions (U, NO_3_, Se, Mn, K, HCO_3_, Zn, I, and F respectively for **a**–**i**) from the deltas are shown. See text for details.

*Potassium (K):* Groundwater generally contains limited amounts of K. In Asian deltas the contribution to the daily intake of K by groundwater is insignificant and, in most cases, less than 10% of RDI (Figure 5e). Similarly, phosphorous (P)—another macro-nutrients, has insignificant presence (compared to RDI of 1000 mg/d) and not included in the analysis.

### 4.2. Status of Micro-Minerals and Toxicants

**Figure 6:**
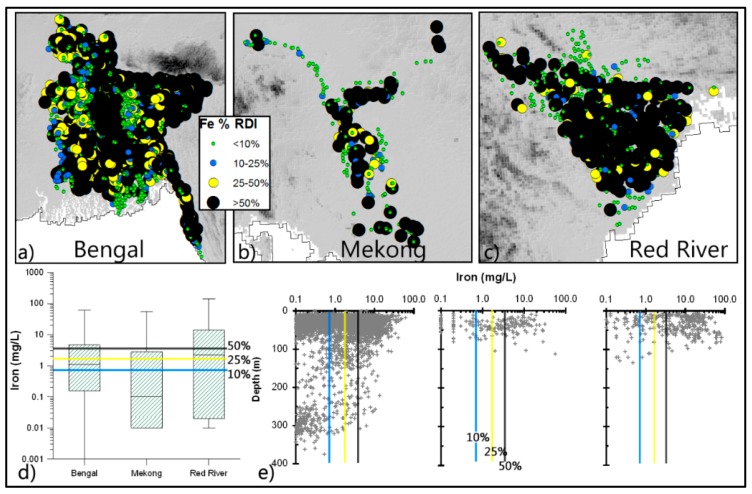
Concentration of Fe in groundwater in relation to RDI. Spatial (**a**–**c**) and vertical (**e**) variations of well-water Fe, and basic statistical properties (as Figure 2) of Fe in well-water are shown in the box plots (**d**).

*Iron (Fe):* Significant amounts of dissolved Fe are present in the analysed groundwaters, with 64% of the wells containing concentrations above 0.3 mg/L, the WHO recommended value, which is for aesthetic reasons and not a health-based guideline for Fe in drinking water. Around 32% of wells in the deltas provide more than 50% of RDI for Fe, while 17% provide more than the daily Fe requirement for an adult. The occurrence of high Fe in groundwater has a regionalised spatial pattern and is relatively higher in shallower aquifers (Figure 6).

*Selenium (Se) and Nitrate (NO_3_):* Selenium is an essential micro-nutrient but in excess concentrations it is harmful for human health. The presence of Se in the groundwaters analysed is largely insignificant and rarely provides anything close to the RDI, although wells in the Red River delta have relatively higher concentrations of Se, but rarely above the 40 µg/L WHO guideline value for drinking water (Figure 5c). These groundwaters are also low in NO_3_, with concentrations well below the WHO guideline value of 50 mg/L (Figure 5b).

*Fluoride (F):* Like Se, a relatively small amount of F is needed for dental and bone health. Although excess F is a problem in some parts of Asia, in the deltas investigated the content of F in groundwater is insignificant. Only a few analyses for F are available for the Bengal delta (although we expect other deltas to be similar) and these indicate that its presence is insignificant, and all are well below the 1.5 mg/L WHO guideline for drinking water (Figure 5i).

*Iodine (I), and Zinc (Zn):* Iodine is widely present in the deltaic groundwaters, with a relatively high concentration in the Red River delta, where half of the wells can provide 50% or more of the RDI (Figure 5h). The presence of Zn in groundwater is insignificant and only in a limited number of cases there is more than 10% of the RDI (Figure 5g).

*Manganese (Mn):* Manganese is an essential micro-nutrient but there is evidence that excessive intake can be harmful [32]. In the groundwaters analysed Mn is present in significant amounts, 44% of wells contain >0.4 mg/L, the WHO’s previous guideline value (which was removed from its recent report as groundwater rarely contains more than 0.4 mg/L Mn [44]). However, these data indicate that around 20% of wells can provide more than 100% of the RDI for Mn. In areas where wells contain significantly high concentrations, these may, when combined with other sources of Mn, result in an exceedance of the RDI for an adult person.

*Arsenic (As):* Groundwater in Asian deltas over the last few decades has been extensively studied for naturally occurring As e.g., [17,19,22,23,45,46,47,48]. Most shallow (*ca.* <100 m) groundwater contains excessive levels of As. In the sets of groundwater analyses for the three delta areas 44% of wells have As concentrations above the WHO guideline, 10 µg/L, for drinking water. The proportion of wells that satisfy the local regulatory standard (all these deltaic countries have the same regulatory value for As in drinking water), of 50 µg/L, is 71% (Figure 1).

*Uranium (U):* Some groundwaters contain U but rarely exceed the WHO guideline value of 30 µg/L.(Figure 5a)

### 4.3. Others

*Bicarbonate (HCO_3_):* Although HCO_3_ is not typically treated as a nutrient, it has some cardio-vascular health benefits [49] and is present in the analysed groundwaters in quantities beneficial for human health. Most wells have concentrations generally >100 mg/L, and typically around 200 mg/L HCO_3_. The concentrations are relatively higher in the Bengal delta, where 50% of wells have >500 mg/L HCO_3_ (Figure 5f).

**Figure 7:**
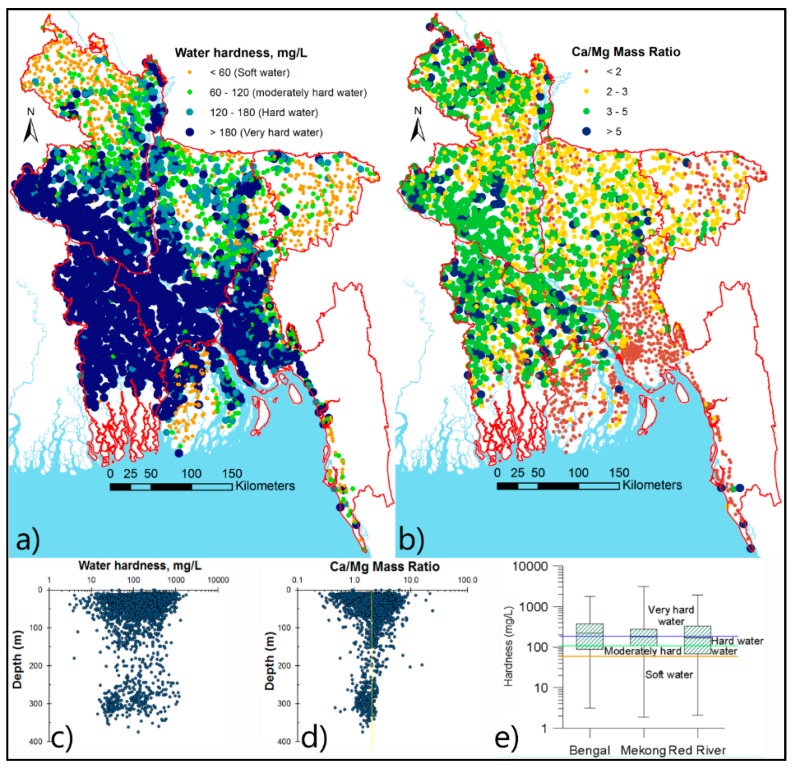
Water hardness and Ca/Mg mass ratio in Bangladesh groundwater. (**a**) Most of the hard or very hard water are found in the southwest region; (**b**) Ca/Mg mass ratio is above 2 most parts except east and southeast region of the country; (**c**) Hardness of water varies irrespective of the well depths; (**d**) Ca/Mg ratio is more homogeneous and around 2 in deeper (>100 mbgl) wells contrary to their shallower depths; (**e**) Box plots indicating hardness of water in other Asian deltas.

*Water hardness and Ca/Mg ratio:* Hardness is caused by the presence of Ca and Mg ions in water and can be calculated as: Hardness = 2.1 × Ca + 4.1 × Mg. Much of the groundwater in Asian deltas is hard *i.e*., >120 mg/L (Figure 7e), and likely beneficial to cardio-vascular health. There is a pattern to the occurrence of hard water, which can be observed in Bangladesh (Figure 7a), where most hard water occurs in the southwest region of the country due to nature of the sediments.

Another important parameter for health reasons is the Ca/Mg ratio, which is reported to be good for cardio-vascular health if the it is 2 or more [50]. In our datasets many groundwaters have a Ca/Mg ratio of 2 or more (Figure 7b).

### 4.4. Potential Hydrogeologically Related Health Impacts (Bangladesh Case Study)

#### 4.4.1. Drinking Water and Diet in Bangladesh

As with many other developing countries, the diet in Bangladesh is highly imbalanced, with rice and other cereals contributing about 80% of total energy. In contrast, fruit and vegetables contribute only 3%, leading to a deficiency in vitamins and minerals in the local population [51]. Protein and micro-nutrient-rich foods like fish, meat, eggs, milk, milk products, fats and oils account for less than 10 percent of a rural person’s diet, and the consumption of vegetables and fruit is declining steadily [52]. However, these assessments do not take into consider the contribution from drinking water. Aside from the coastal areas, over 97% of the national population in Bangladesh relies on groundwater as a source of potable water. As demonstrated in the previous section, for some nutrients, (e.g., Ca, Mg, Fe), more than 50% of the RDI can be obtained by drinking two litres of water and can, therefore, potentially provide a significant contribution where dietary intake (e.g., Ca, Mg and Fe) is below the recommend level [53].

#### 4.4.2. Possible Link between Mg and Low Birth-Weight and Diabetes

**Figure 8:**
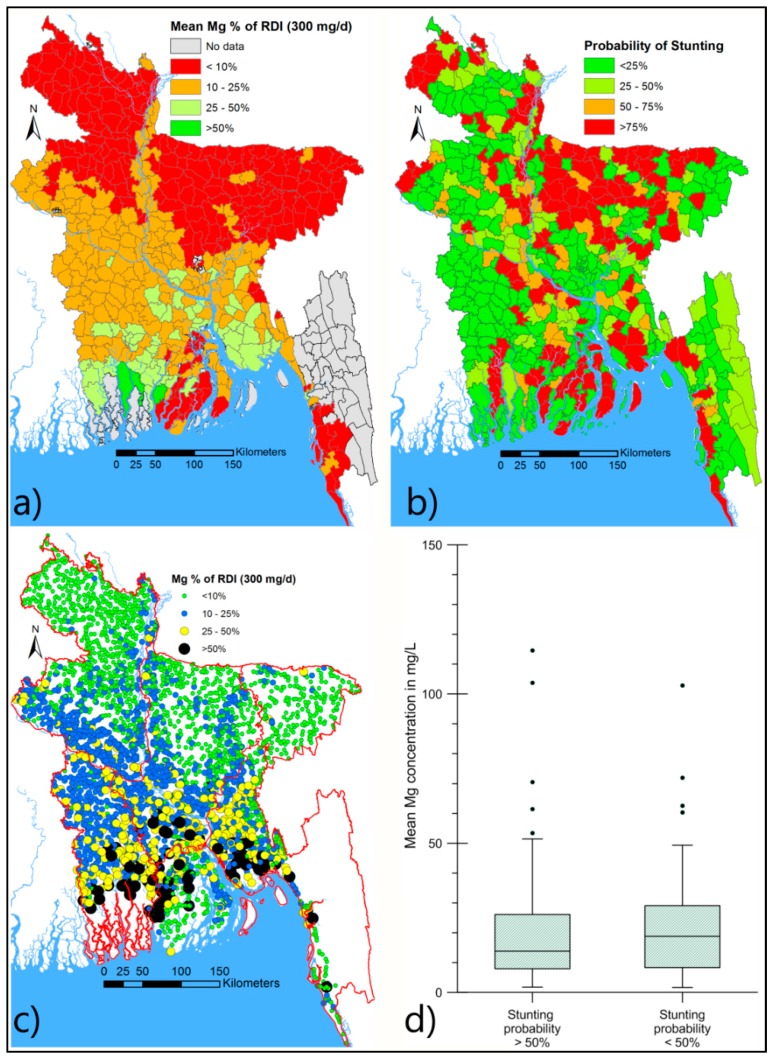
The possible association between tubewell Mg concentration and probability of under-five children stunting in Bangladesh. (**a**) upazila based mean Mg concentration [22] in relation to RDI; (**b**) upazila based probability of stunting in children under-five in Bangladesh (from http://www.foodsecurityatlas.org, accessed 1 August 2014); (**c**) Spatial variation of Mg in well water in Bangladesh; (**d**) mean Mg concentration in upazilas (panel—**a**) where probability of stunting (panel—**b**) is > 50% and < 50%.

Despite considerable improvement in national health status, children and women in Bangladesh suffer from high levels of malnutrition and micro-nutrient deficiencies [54,55]. This leads to low birth weight (currently 30%–50% of newborns are underweight), stunting (*ca.* 50%), and wasting (*ca.* 12%) in under-five year old children (www.foodsecurityatlas.org/bgd/country, accessed 1 August 2014). These underweight children are susceptible to chronic diseases in later life. Magnesium deficiency during pregnancy can lead to low birth weight [56,57]. In Bangladesh the distribution of Mg in tubewells and stunting in children under-5 years show good spatial correlation (Figure 8), and comparison with the spatial distribution of low birth-weight (not shown) is also similar.

Deficiency in Mg during pregnancy, as well as in adulthood has been linked to glucose intolerance potentially leading to type 2 diabetes [58,59]. The overall, age-adjusted prevalence of diabetes (largely a lifestyle related health condition) and pre-diabetes in Bangladesh is 10% and 22% respectively, with significant spatial variation of prevalence [60]. In Bangladesh spatial differences in tube well Mg concentrations are also significant (Figure 8). However, the influence of this spatial variation on the spatial pattern of prevalence of diabetic and pre-diabetic conditions has yet to be established. The association has not been tested or explored elsewhere, and if a link exists, its recognition would help improve our understanding of the spatial prevalence of diabetes, which could help formulate appropriate interventions.

#### 4.4.3. Possible Role of Ca in Bone Health, and Joint Role of Ca and Mg in Blood Pressure Regulation

As well as a nutrient for bone health (along with vitamin D), Ca has also been (in association with Mg) linked with hypertension by number of previous studies e.g., [50]. The Ca and Mg intake of much of the Bangladeshi population does not meet daily requirements [61,62].

**Figure 9:**
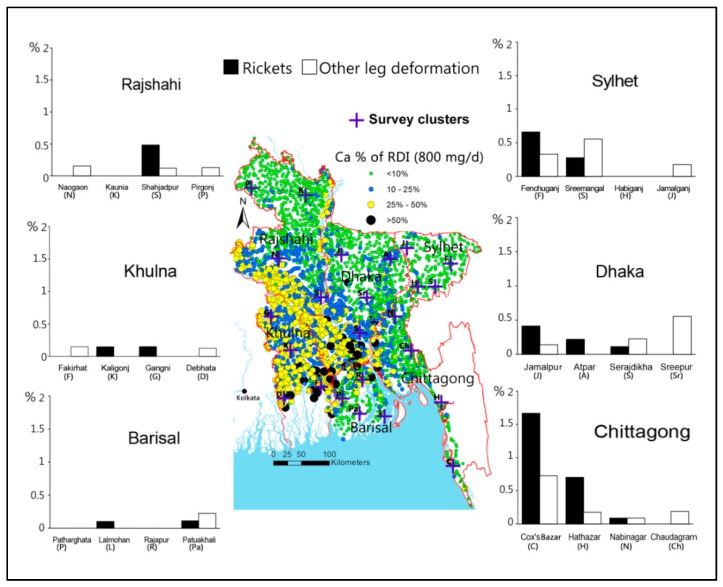
Possible spatial association between prevalence of rickets and other leg deformities and Ca concentration in tubewells in Bangladesh. Water composition data from [22], and data on rickets and other leg deformities are based on [64].

The widespread nature of “Ca undernutrition” almost certainly contributes to the emergence of rickets in Bangladesh and the prevalence of osteoporosis [63]. In south-east Bangladesh, rickets caused by Ca deficiency appears to affect some 4% of children. A separate survey in several sub-districts indicates a tentative spatial association with the well water Ca (Figure 9). Dietary intake, estimated from dietary surveys, contribute around 200 to 400 mg/d of Ca, which is generally less than 50% of the RDI [53,61]. However, if food habits are assumed to be similar throughout the country then it might be anticipated that in some areas people should not suffer from Ca deficiency because around 50% of the RDI is contributed by drinking water in those areas. Bangladesh is a sunny country where many people spend a considerable amount of time outdoors with opportunities to produce adequate vitamin D. The two most important nutrients for bone health are vitamin D and Ca and in the presence of sufficient vitamin D, Ca deficiency may be actively influencing bone health in the population of Bangladesh.

Previous studies have shown that hard water (high in Ca and Mg, and with a Ca/Mg ratio of 2 or more) is good for blood pressure regulation in the body, and soft-water has been linked with cardiovascular diseases see [50]. In Bangladesh, the spatial distribution of Ca and Mg clearly shows higher concentrations in the south-west compared to other regions. Mapping of water hardness indicates that the presence of several zones of distinct water hardnesses in the country (Figure 7) but no study has been done to test the hypothesis of association between cardiovascular diseases and water hardness.

#### 4.4.4. Anaemia and the Tubewell Fe Content in Bangladesh

**Figure 10:**
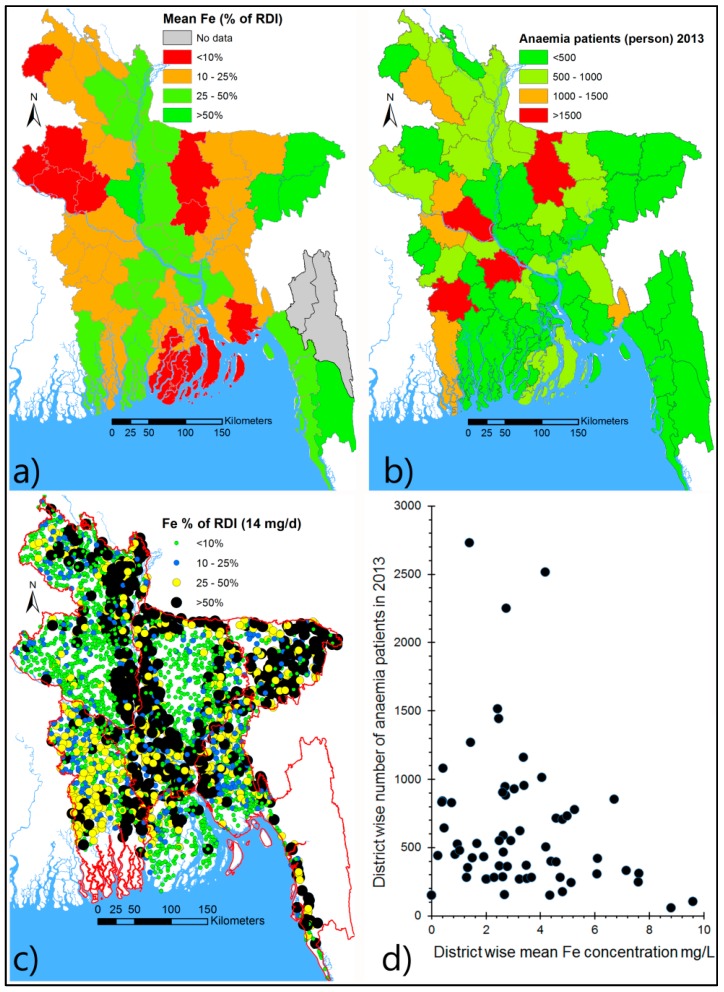
Possible inverse relationship between Fe concentration in tubewell water and anaemia in Bangladesh. (**a**) District based mean Fe concentration [22] in relation to RDI; (**b**) district based anaemia patients attended in hospital in 2013,see text for data source; (**c**) spatial variation of well-water Fe in Bangladesh; (**d**) scatter plot of district based mean Fe (panel—**a**) concentration and district based number of anaemia patients (panel—**b**) in 2013.

In Bangladesh >50% of pre-school children, women of reproductive age and adolescents suffer from Fe deficiency-related anaemia. Iron deficiency in Bangladesh should be obvious if only food intake is considered [53] but most tubewell water contains significant amounts of Fe (occasionally around 100% of RDI or more, Figure 10). However, drinking water may completely lack folate—also important in preventing anaemia. Elements (e.g., As) that are detrimental to health also exist in wells that have higher Fe concentrations e.g., [16]. A preliminary analysis of district wide anaemic patients (who attended hospitals in 2013, http://www.dghs.gov.bd, accessed on 5 August 2014) indicates that the numbers of patients are low in areas where tube wells have a higher Fe concentration (Figure 10). The analysis is not adjusted for age or sex and neither does it consider other confounding factors due to lack of information. Bio-availability of the Fe in drinking water is also an undetermined factor, which depends on types of consumed food, and method used for water storage after collection. Previous studies in Bangladesh [65,66] indicate significant absorption of Fe by the human body can be derived from drinking water.

#### 4.4.5. Spatial Distribution of As and Arsenicosis in Bangladesh

**Figure 11:**
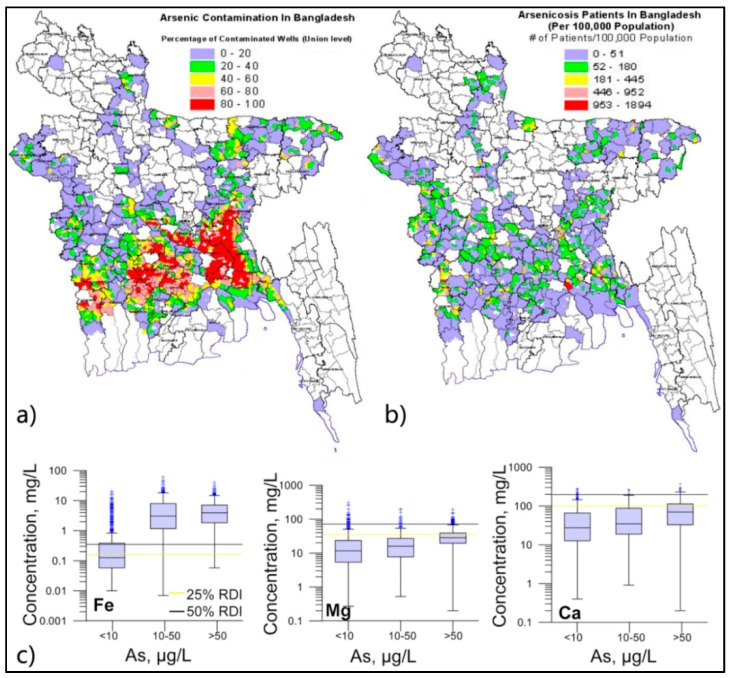
Spatial variation of well water concentration of As and arsenicosis patients in Bangladesh [67]. (**a**) Percentage of tubewells contain As concentration, above 50 µg/L—Bangladesh standard for drinking water As, at union level; (**b**) distribution of arsenicosis patients in Bangladesh. The patients’ data have a number of limitations, in particular the influence of beneficial mineral nutrients on patients is not known; (**c**) High As wells in Bangladesh indicate higher concentrations of Fe, Mg and Ca.

The Bangladesh Arsenic Mitigation Water Supply Project (BAMWSP) has undertaken field screening of around 5 million tubewells. This shows that, of the wells tested, 29% contained As above the Bangladesh standard of 50 µg/L [67]. The BAMWSP then prepared a map using all the screening data obtained from different health organizations on occurrence of arsenicosis patients (*n* = 38,430) located in different areas of the county (Figure 11). Although there are some concerns over the validity of the health data, there appears to be a spatial relationship between As contaminated areas and presence of arsenicosis patients (Figure 11).

## 5. Discussion

### 5.1. Coexistence of Nutrients and Toxicants in Groundwater

The components in drinking water are generally analysed in order to avoid passing on chemicals harmful to human health. In contrast, little, if any, attention is given to constituents beneficial to health. WHO guideline values for drinking water reflect this and are essentially set for toxic elements, along with micro-nutrients that are essential at certain concentrations but become a health problem when in excess [68]. No guideline values are provided for essential macro-nutrients, except for aesthetic purposes (e.g., the limits for Cl and Na are set at 250 and 200 mg/L, respectively, as concentrations above these levels make the water taste salty). Most groundwater in Asian deltas is rich in mineral nutrients (e.g., Ca, Mg, Fe) occasionally providing >50% of the RDI. This drinking water contribution varies spatially as individually managed tubewells are distributed over a large region and groundwater composition varies.

Although the groundwaters sampled contain some beneficial minerals, it is also found, for example, that wells containing high levels of Fe also may contain excessive levels of dissolved As. In addition, some of these wells also contain beneficial levels of Ca and Mg, thus further complicating the situation (Figure 11c). Human health studies have shown that As in drinking water is often linked to an increased risk of cardiovascular diseases [69], whereas other such studies have shown that the presence of Ca and Mg, with a Ca/Mg ratio of around 2, is better for cardiovascular health [50]. The impact on human health of these juxtaposing conditions is not currently known, however studies on rats have shown that the presence of high Ca and Mg levels can reduce the toxicity of As [70].

Iron is the single micro-nutrient whose deficiency affects >2 billion people in the world and a great majority of these people are living in developing countries (http://www.who.int/nutrition/topics/ida/en/, accessed on 20 August 2014). Most Asian deltaic groundwaters contain significant amounts of Fe, although the bioavailability of this iron is not fully understood [42]. However, preliminary studies [65,66] in Bangladesh have shown a significant absorption of drinking water Fe by human body. Iodine is an important micro-nutrient [71] and a significant part of RDI appears to be provided from drinking groundwater in these deltaic regions. Other important nutrients, e.g., F and Se, are not present at a concentration beneficial for human health and, in a similar way, some potentially toxic substances, e.g., U and NO_3_, are at concentrations too low to have any serious impact on human health.

**Figure 12:**
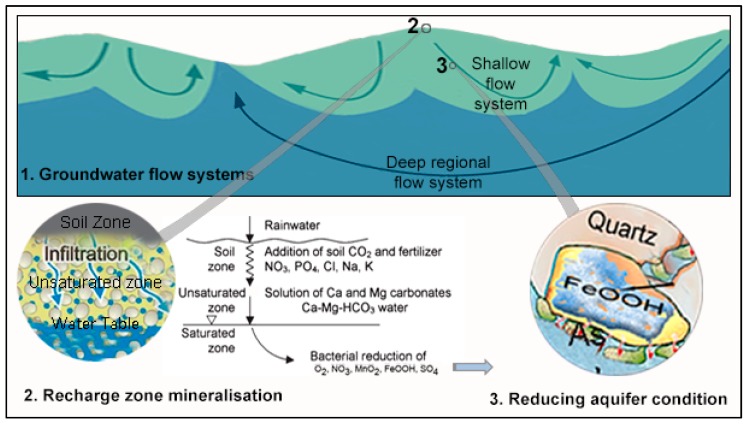
Conceptual illustration of main controls and processes on water chemistry (recharge zone mineralisation [72], and microbial mediated redox reactions [73,74,75] driven by the biodegradation organic matter and involve the consumption of oxygen, nitrate, manganese- and iron-(hydro)oxides, and also sulfate) in Asian deltaic aquifers influencing groundwater quality and nutrient contents.

Although Na is a macro-nutrient, its intake is generally considered only in terms of that derived from food, along with added salt for flavour. However, some groundwaters in coastal areas provide significant amounts of Na when used as a source of drinking water, and, when combined with food derived Na, can result in levels of Na intake exceeding the WHO recommended daily amount of 2 g/d. This extra Na can lead to high blood pressure and hypertensive disorders in some local populations [33,34]. The high levels of Ca and Mg in this deltaic groundwater are often accompanied with high levels of HCO_3_. Studies have shown that high HCO_3_ in drinking water may counter the impacts on blood pressure due to excess Na [76,77], although it is not currently known whether this is the case for the coastal populations in living in Asian deltas.

### 5.2. Medical Hydrogeology: Controls on Groundwater Nutrients and Toxicants

Geological history, hydrogeology and aquifer geochemical processes determine what kind of mineral nutrients and toxicants can be expected in an aquifer e.g., [78]. In terms of its effects on human health, an aquifer can be characterised in terms of the presence or absence of certain nutrients and toxicants. Such a characterisation may, in turn, help both individuals and/or health professionals to appreciate the contribution from groundwater to an individual’s nutrition and risk from toxicants. There are three main processes that affect this: recharge, geochemical processes, including ion-exchange and the differentiation of groundwater flow into shallow and deep systems (Figure 12). Like many other aquifers, these processes in the Asian deltaic aquifer lead to the presence of some nutrients and toxicants and the absence of others.

Near surface groundwater is generally infiltrated rainwater that has reached the water table through an unsaturated zone. The recharge water undergoes quality changes both during infiltration and then during its movement through the subsurface. Rain water is generally low in mineral content, however, the addition of soil carbon dioxide and mineral fertiliser (which are widely used in these areas) lead to carbonate dissolution along with silicate weathering and therefore increased levels of Ca, Mg, HCO_3_, PO_4_, NO_3_, Cl, Na, and K. The major ion chemistry indicates that much of the groundwater has acquired its chemical composition during recharge as the infiltrating of rainwater is transformed into Ca-HCO_3_ type water [79].

The high organic content within these aquifers help drive microbial reduction, which removes any infiltrating dissolved oxygen and NO_3_ from the recharging water, leading to the release of Mn and Fe and, consequently, the release of adsorbed As on MnOOH and FeOOH minerals into solution [79]. Among the deltas investigated, the Red River delta has relatively high concentrations of Fe (Figure 6) because of strong reducing conditions along with relatively lower pH and HCO_3_ [80]. The reducing environment also keeps U, Se and V, where present in sediments, in solute form. Although some U and V have also been found in the brown sand aquifers, where the aquifer zone is slightly oxic [36], they are below WHO guideline values. These brown sand aquifers are also relatively low in Ca, Mg and slightly higher in Na compared to their grey reduced counterpart. The enrichment of Ca may also be keeping F concentrations low. In addition to redox reactions, there are also ion exchange and precipitation processes, which lead to changes in ion concentrations along the flow path. In coastal areas, the high Na concentrations found in aquifers are associated with poor flushing of the syndepositional salinity [81]. The different groundwater flow-systems play an overarching role in chemical concentrations. With relatively high nutrient loads and other anthropogenic pollutants in shallower aquifers (*i.e.*, within *ca.* 100 bgl). Deeper groundwater, which is typically a few thousand years old [82] rarely contains anthropogenic pollutants. In addition, most of the As contaminated wells are tapping water from the shallow flow-system operating at a depth shallower than *ca.* 100 m.

### 5.3. Health Benefit in Resource Poor Settings and Food Frequency Questionnaire

Where intake through food is insufficient, the addition of nutrients contained in drinking water may help those in such circumstances attain the daily required intake. Our exploratory research in Bangladesh indicates that the levels of nutrients in groundwater is so substantial that in some areas individuals can obtain up to 50% or more of the RDI (e.g., Ca, Mg, Fe) from just two litres of drinking water. Consumption of groundwater is therefore promoting better heath in some areas, whilst in other areas the lower nutrient content in groundwater may contribute to the prevalence of non-communicable diseases including anaemia, hypertension, glucose intolerance (diabetes), rickets and bone deformation.

In view of the contribution that groundwater can provide to nutrient intake, this implies that an understanding of the total nutritional level of an individual in such cases cannot be properly determined without considering the contribution of the nutrients and minerals supplied by drinking water. However, there is no known protocol to incorporate drinking-water-derived nutrients in the framework of dietary assessment, and existing dietary assessments do not take into consideration the contribution from drinking water [83].

There are no health-based guidelines for beneficial nutrients, though Rosbborg [68] has suggested one based on the analysis of ionic balance of a large number of samples that may be used in a centrally managed water supply system. Pragmatically, it would not be possible to implement such a scheme in Asian deltas as most of the water sources *i.e.*, tubewells are distributed over a large area with heterogeneous water quality and individually managed.

This paper has demonstrated the potential health benefits of drinking water nutrients for those with nutrient poor diets, particularly in the case of calcium, magnesium and iron, using limited demographic health data from Bangladesh. The concentrations of beneficial nutrients in many areas of this and other deltas are such that these probably minimize the daily intake gap between the RDI and that obtained from the food. The results indicate a need for further studies with more rigorous quality-controlled health data to evaluate the health benefits. We anticipate the health benefit of groundwater would be similar to Bangladesh in other deltas and should be explored.

## 6. Conclusions

From this exploratory work we would like to put forth the following conclusions:

- Drinking groundwater may supply substantial amounts of nutrients beneficial to human health, even though their adsorption by the body and overall health benefits are yet to be fully determined.
- In addition, groundwater may also contain toxicants and therefore coupling or decoupling the toxicity with other constituents present in the water needs to be studied in an holistic manner.
- An understanding of the association of non-communicable disease and poor nutrition cannot be developed, particularly in areas with high levels of dissolved solids in water sources, without considering the contribution of drinking water to nutrient and mineral supply.
